# Variation and stability of rhizosphere bacterial communities of *Cucumis* crops in association with root-knot nematodes infestation

**DOI:** 10.3389/fpls.2023.1163271

**Published:** 2023-05-30

**Authors:** Liqun Song, Xingxing Ping, Zhenchuan Mao, Jianlong Zhao, Yuhong Yang, Yan Li, Bingyan Xie, Jian Ling

**Affiliations:** ^1^ Insititute of Vegetables and Flowers, Chinese Academy of Agricultural Sciences, Beijing, China; ^2^ State Key Laboratory of Vegetable Biobreeding, Institute of Vegetables and Flowers, Chinese Academy of Agricultural Sciences, Beijing, China; ^3^ Microbial Research Institute of Liaoning Province, Liaoning Academy of Agricultural Sciences, Chaoyang, China

**Keywords:** time dynamic, co-occurrence network, rhizosphere bacteria, Cucumis crops, root-knot nematodes

## Abstract

**Introduction:**

Root-knot nematodes (RKN) disease is a devastating disease in *Cucumis* crops production. Existing studies have shown that resistant and susceptible crops are enriched with different rhizosphere microorganisms, and microorganisms enriched in resistant crops can antagonize pathogenic bacteria. However, the characteristics of rhizosphere microbial communities of *Cucumis* crops after RKN infestation remain largely unknown.

**Methods:**

In this study, we compared the changes in rhizosphere bacterial communities between highly RKN-resistant *Cucumis metuliferus* (cm3) and highly RKN-susceptible *Cucumis sativus* (cuc) after RKN infection through a pot experiment.

**Results:**

The results showed that the strongest response of rhizosphere bacterial communities of *Cucumis* crops to RKN infestation occurred during early growth, as evidenced by changes in species diversity and community composition. However, the more stable structure of the rhizosphere bacterial community in cm3 was reflected in less changes in species diversity and community composition after RKN infestation, forming a more complex and positively co-occurrence network than cuc. Moreover, we observed that both cm3 and cuc recruited bacteria after RKN infestation, but the bacteria enriched in cm3 were more abundant including beneficial bacteria Acidobacteria, Nocardioidaceae and Sphingomonadales. In addition, the cuc was enriched with beneficial bacteria Actinobacteria, Bacilli and Cyanobacteria. We also found that more antagonistic bacteria than cuc were screened in cm3 after RKN infestation and most of them were *Pseudomonas* (Proteobacteria, Pseudomonadaceae), and Proteobacteria were also enriched in cm3 after RKN infestation. We hypothesized that the cooperation between Pseudomonas and the beneficial bacteria in cm3 could inhibit the infestation of RKN.

**Discussion:**

Thus, our results provide valuable insights into the role of rhizosphere bacterial communities on RKN diseases of *Cucumis* crops, and further studies are needed to clarify the bacterial communities that suppress RKN in *Cucumis* crops rhizosphere.

## 1 Introduction

Nematodes were reported to be the second most numerous in the animal kingdom residing in multiple ecosystems, with up to one million species (Bongers and Bongers, 1998; Mitreva et al., 2005). Root-knot nematodes (RKN) were among the top ten plant parasitic nematodes in the world found in almost all vascular plants, causing severe crop losses through direct injury and transmission of pathogenic microorganisms (Chalivendra, 2021). The most devastating RKN species were *M. incognita*, *M. arenaria*, *M. hapla* and *M. javanica*, causing hundreds of billions of dollars in crop losses each year (Nicol et al., 2011; Singh et al., 2015). Variety of the plant affected their response to RKN parasitism, which usually induced knotted gall tumors in the host plant (Jones et al., 2013). RKN penetrated root cells and induced the production of giant cells near the vascular bundle, from which nematodes to absorb nutrients by mouthparts (Moens et al., 2009).

Cucumber (*Cucumis sativus*), an economically important crop, in the Cucurbitaceae family is widely grown around the world (Kaur et al., 2023). The RKN was one of the most important diseases in cucumber (*Cucumis sativus*) growing regions around the world. It was estimated that RKN caused yield losses of 88% in cucumbers under greenhouse cultivation conditions (Expósito et al., 2019). The ubiquitous RKN developed different lifestyles and feeding strategies, which, made them difficult to manage (Topalović and Vestergård, 2021). Chemical nematicides have been widely used to control plant-parasitic nematodes, but the harmful effects of these chemicals on the environment have led to a significant reduction in their frequency of use. Currently, host plant resistance was an effective method for managing plant-parasitic nematodes, but in some cases, high genetic diversity within and between nematodes populations confounded the use of available resistant species and limited their effectiveness (Li et al., 2011). To date, there was no cultivated cucumber cultivars with RKN resistance (Weng, 2010). In contrast, a high resistance to RKN were found in African horn cucumber (*Cucumis metuliferus*), a relative of African-endemic *Cucumis* (Walters et al., 2006). The *C. metuliferus* was a member of the Cucumoraceae, which included several economically important crops such as cucumber, melon, squash and watermelon (Nakata et al., 2005). It was a potential genetic resource for Cucumoraceae crops improvement as it contains genes for resistance to a variety of Cucumoraceae crops pests and diseases (Yagi et al., 2014). And it has been reported to be resistant to RKN, gummy stem blight and *Fusarium* wilt (Chen et al., 2020). In this case, resistance to RKN in *C. metuliferus* was associated with reduced nematode penetration, developmental retardation and hypersensitive necrosis (Ye et al., 2017). However, the attempt of interspecific hybridization between *C. metuliferus* and *C. sativus* was not successful (Walters et al., 2006).

Biological control was a means of suppressing pests and pathogens by using other organisms that can be natural enemies, such as predators, parasitic organisms and competitors, with the advantages of environmental safety and precise targeting, of which microorganisms were receiving increasing attention (Vega, 2018; Chalivendra, 2021). Microorganisms that colonize plants in numbers that far exceed the number of plant cells were called the plant microbiome, which played an important role in plant growth and development (Mendes et al., 2013). There were several interaction ways between microorganisms and nematodes. Microbial competition for space, nutrients and water reduced nematode activity and reproductive capacity (Pal and McSpadden Gardener, 2006). And microorganisms produced and released certain antibiotics or toxins which may adversely affect the nematode during the infection stage (Lugtenberg and Kamilova, 2009). Many bacterial products can induce systemic resistance in plants to protect the whole plant from pathogenic bacteria and nematodes (Junaid et al., 2013). Some bacteria can also penetrate the cuticle and killed nematodes by the action of enzymes (Tian et al., 2007). In addition, microorganisms contributed to plant development by improving the solubility, uptake and absorption of nutrients, thus helping to increase the tolerance of plant roots to plant-parasitic nematodes (Schouteden et al., 2015). Many antagonistic microorganisms, including *Purpureocillium lilacinum*, *Streptomyces*, *Pseudomonas*, have been tested and widely used to suppress a number of plant pathogens and nematodes (Sarwar et al., 2018; Aiello et al., 2019; Jiao et al., 2019).

The assembly of the microbiome included dynamic changes in species composition and abundance, on the other hand, the steady-state composition of spatially distinct compartments. The rhizosphere, the narrow area around and affected by plant roots, was an important ecological niche in plant microbiome studies and one of the most complex ecosystems on Earth (Hinsinger et al., 2009; Raaijmakers et al., 2009). “Rhizosphere effect”, a phenomenon in which the rhizosphere microbial community differed from the community in the bulk soil, implied that plant roots recruited specific microorganisms including nitrogen-fixing bacteria, biocontrol microorganisms and protozoa, plant-promoting rhizobacteria (PGPR) from the bulk soil to the rhizosphere (Hein et al., 2008; Mendes et al., 2013). The interactions between these rhizosphere microorganisms that were beneficial to plant growth can impact their effects. In addition, the rhizosphere was the area where roots and their exudates affected various biological and ecological processes by interacting with microorganisms (Fang et al., 2013). Microbial interactions, complexity and diversity were of considerable importance to the formation and homeostasis of microbial communities (Cordovez et al., 2019). Also, microbial composition can be influenced by plant genotype, developmental stage (Bulgarelli et al., 2012; Zachow et al., 2014), fertilization management (Saha et al., 2008) and soil type (Schlaeppi et al., 2014).

Infestation by pathogens or RKN can disrupt the original rhizosphere microbial community structure of the crop and alter microbial diversity. A previous study of the *Arabidopsis* microbiome showed that plants can specifically recruit a set of beneficial microbes that induce resistance and promote growth in response to pathogen infection (Berendsen et al., 2018m). Besides, the Flavobacterium dominated the rhizosphere of resistant tomatoes after *Ralstonia solanacearum* infestation and tested that Flavobacterium could antagonise *Ralstonia solanacearum* (Kwak et al., 2018). The bacterial wilt outbreaks modified microbial composition and diversity as well as reduce the abundance of beneficial microorganisms in the soil (Wang et al., 2017). *Fusarium* wilt-diseased banana had a higher abundance and diversity of fungi or bacteria than disease-free soils, showing a change in the dominant phylum (Zhou et al., 2019). A study showed that tobacco composition of the root microbial community was significantly associated with RKN infection (Cao et al., 2022). In contrast, variations in the rhizosphere microbial community resulting from RKN infestation of *Cucumis* crops remain unclear.

Previous studies showed that *C. metuliferus* had the highest number of disease-resistance-associated NBS-LRR genes in Cucurbitaceae by assembling and analyzing the chromosomal level genome of *C. metuliferus* (Ling et al., 2021), and that root volatiles of *C. metuliferus* had potential applications against RKN (Xie et al., 2022), which implied the resistance to RKN in *Cucumis* crops was a complex and integrated mechanism. Therefore, this study compared and analyzed the response of rhizosphere bacterial community to RKN infestation in resistant *C. metuliferus* and susceptible *C. sativus*.

## 2 Materials and methods

### 2.1 Pot experiment and sample collection

*C. sativus* inbred line 9930 (cuc) and *C.metuliferus* inbred line CM3 (cm3) were provided by Institute of Vegetable and Flowers, Chinese Academy of Agricultural Sciences (IVF-CAAS; Beijing, China). The *M. incognita* used in this study was obtained from the Institute of Vegetables and Flowers, Chinese Academy of Agricultural Sciences (IVF-CAAS; Beijing, China). The soil with a multi-year history of nematodes infestation was sampled from a greenhouse in Langfang city (Hebei, China). Then, the plants were maintained in a sterile mixture of soil: vermiculite: perlite (2:2:1, vol/vol/vol) in a glass room of a phytotron in April 2021 with day/night temperatures 28/18°C.

*M. incognita* was inoculated on the roots of the hollow cabbage. Approximately 45 days after inoculation, the *M. incognita* egg masses were picked with forceps and disinfected with 0.5% NaClO for 15 seconds. The egg masses were rinsed several times with sterile distilled water so that no NaClO remained on the surface, then placed in a sterile Petri dish with 20 mL of sterile water and incubated in a light-proof incubator at 28°C. The second-stage *M. incognita* juveniles (J2s) were counted using a stereomicroscope for bioassays.

The cuc and cm3 seeds were soaked in 0.5% NaClO for 2-3 minutes and then rinsed several times with sterile distilled water until no NaClO remained. The surface sterilised seeds were laid flat in a petri dish with sterile filter paper, soaked with a small amount of sterile distilled water (not over the seeds) and incubated in the dark at 28°C in an incubator. The seedling substrate and vermiculite were sterilised twice in an autoclave (121°C, 60 min) and the germinated seeds were sown into the sterilised substrate, which consisted of grass charcoal soil and vermiculite in a 2:1 volume ratio. Then the seedling cavity trays were grown in a greenhouse to obtain soil-grown seedlings for subsequent experiments.

The experiment consisted of five experimental groups, cm3, cuc, cm3 inoculated with nematodes (cm3J), cuc inoculated with nematodes (cucJ) and bulk soil. One sample (rhizosphere soil mix of five randomly selected plants) from each treatment at a time was taken and five replicates were set up. Each plant was inoculated with 500 *M. incognita* in cm3J and cucJ groups. Approximately 30 days (T1) and 60 days (T2) after inoculation, each plant was uprooted to prepare for rhizosphere samples. The roots without large pieces of soil were placed in a 250 ml sterilized conical flasks with phosphate buffer saline (pbs) buffer submerged. The conical flasks were placed on a shaker (20 min, 25°C, 160 r) to remove the soil still attached to the roots. Afterwards, the conical flasks were left to stand in a refrigerator at 4°C for 24 h. Finally, the lower soil solution was collected as the rhizosphere sample. In total, 40 rhizosphere and 10 bulk soil samples were collected.

### 2.2 DNA extraction and 16S ribosomal RNA gene amplification

Total bacterial genomic DNA from 0.5 g of soil for each sample was extracted using a Fast DNA SPIN extraction kit (MP Biomedicals, Santa Ana, CA, USA) according to the manufacturer’s instructions. The DNA was stored at -20°C until further analysis. The quantity and quality of extracted DNA was measured using a NanoDrop ND-1000 spectrophotometer (Thermo Fisher Scientific, Waltham, MA, USA) and agarose gel electrophoresis, respectively. The V3-V4 region of bacterial 16S rRNA genes was amplified by PCR using the forward primer 515F (5’-GTGCCAGCMGCCGCGGGTAA-3’) and the reverse primer 907R (5’-CCGTCAATTCMTTTRAGT1T-3’). The primers were incorporated into sample specific 7 bp barcodes for multiplex sequencing. After individual quantification, equal amounts of amplicons were pooled and pair-end 2x300 bp sequencing was performed on the Illumina MiSeq platform using the MiSeq Reagent Kit v3 at Shanghai Personal Biotechnology Co., Ltd (Shanghai, China). The 16S sequence datasets for this study can be found in the NGDC with accession number: PRJCA014808 (https://ngdc.cncb.ac.cn).

### 2.3 Bioinformatic and statistical analysis

A total of 5,262,484 raw reads were obtained from 50 samples, with sequence lengths greater than 400 bp. After splicing the raw reads, double-ended primers were excised, and low-quality reads with a Q scores less than 30 were filtered. Operational taxonomic units (OTU) were clustered using usearch (v10.0) with a 97% similarity cutoff, and chimeric sequences were identified and removed using vsearch (v2.8) based on gold.fa (http://drive5.com/uchime/rdp_gold.fa). Each bacterial 16S rRNA gene was annotated in the greengenes database (gg_16s_13.5) using vsearch (v2.8). The OTU without annotation and annotations for Chloroplast, Mitochondria, Archaea were deleted. Bacterial reads were classified into 4531 OTU after quality filtering. Then, the OTU with relative abundance less than 0.001% were filtered and flattened according to sample_size 52353. We calculated bacterial alpha diversity using the Shannon indices and observed_otus by vsearch (v2.8), and bacterial beta diversity using principal co-ordinates analysis (PCoA) based on the Bray–Curtis dissimilarity matrix by edgeR and vegan packages. Data on the relative abundance differences between groups of bacteria at the phylum level were processed with the dplyr and reshape2 packages. The unique and shared OTU of different groups were plotted by the ggVennDiagram package (Gao et al., 2021). The volcano plot drawn with the edgeR and ImageGP packages showed OTU enrichment differences between groups. Bacterial co-occurrence networks of different groups at different stages visualised correlations with |RHO| > 0.9 and P < 0.05 using the ggCLusterNet package (Wen et al., 2022). Stamp, linear discriminant analysis effect size (LEfSe) analysis and circos plot were performed using the OmicStudio tools at https://www.omicstudio.cn/tool.

### 2.4 Isolation and screening of nematodes resistant bacteria

Samples from each group were mixed and enriched for bacteria by the following steps. 1) 100 ml of soil solution was added to 150 ml of pre-cooled distilled water, then slowly filtered through a double layer of gauze after mixing well. 2) The supernatant obtained by centrifugation of the filtered solution (700 r, 4°C, 5 min) was collected. 3) The precipitate was resuspended and centrifuged twice more, and the supernatants of the three times were combined. 4). After centrifugation (1000 r, 4°C, 10 min) of all the supernatants, the precipitate was resuspended with 15 ml of 0.8% NaCl. 5) The resuspension was centrifuged (11000 r, 4°C, 30 min) after the slow addition of 10 mL of Nycodenz (0.8 g/mL). 6) After centrifugation, it was divided into Nycodenz-soil mixed particles (lower layer), water (upper layer), and bacteria (middle layer). 7) The bacterial layer, resuspended by the addition of 10 mL of sterilised distilled water, was centrifuged (10,000 r, 4°C, 20 min) to obtain the Nycodenz-free bacteria.

The enriched bacteria were cultured on LB and R2A medium plates, and 20 single colonies of different sizes and morphologies were picked from each plate. Then 600 single colonies were obtained in 3 replicates. The nematicidal activity of the strains was tested in a 24-well plate. The 900 μL of bacterial broth was mixed with 100 μL of nematodes suspension (approximately 100 *M. incognita*) in individual wells. Additionally, 100 μL of nematodes suspension (approximately 100 *M. incognita*) mixed with 900 μL of sterilised distilled water and culture medium respectively were added to other wells to serve as untreated controls. The 24-well plates were incubated at 28°C for 24 h. Nematodes were considered dead if the bodies of nematodes were straight and did not move when stimulated with 0.5 M NaOH (Harada and Yoshiga, 2015). The corrected mortality rate was reference Yin (Yin et al., 2021). Bacteria with the corrected mortality rate greater than 85% compared to the corresponding medium were considered as antagonistic bacteria. Genomic DNA of strains were extracted using the TIANamp Bacteria DNA Kit (Tiangen, Beijing China) and the 16S ribosomal DNA was amplified by PCR using 2x Rapid Taq Master Mix (Vazyme, Nan- jing China) and primers 27F and 1492R (Alberoni et al., 2019). The following conditions were used: denaturation at 95°C for 3 min; followed by 34 cycles at 95°C for 30 s, 55°C for 30 s, and 72°C for 1 min; and extension at 72°C for 5 min. The product was stored at 4°C until sequencing. The sequence results were analyzed using the NCBI BLAST tool (https://www.ncbi.nlm.nih.gov/).

## 3 Results

### 3.1 Diversity and abundance of bacterial communities in different groups

**Figure 1:**
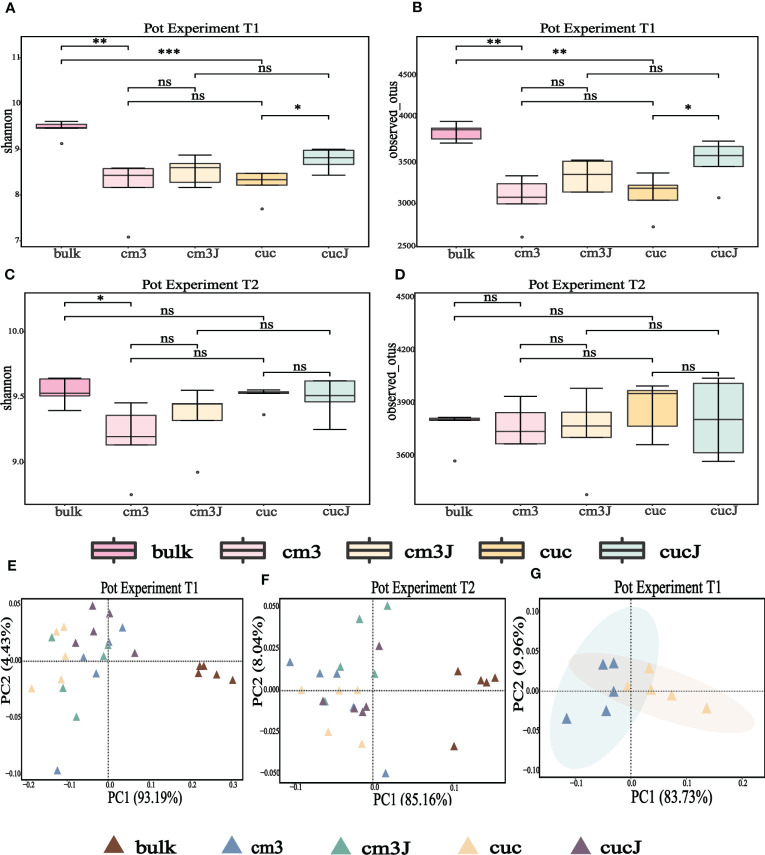
Alpha diversity (shannon indices and observed OTU) of bacteria in the rhizosphere at T1 **(A, B)** and T2 **(C, D)**. Differences between the cm3, cuc, cm3J, cucJ, and bulk soil were indicated in each figure panel (ns p> 0.05, *p < 0.05, **p < 0.01, and ***p < 0.001). Principal co-ordinates analysis (PCoA) analysis of bacteria at T1 **(E, G)** and T2 **(F)** in the rhizosphere of different group on Bray–Curtis distance metrics.

At the operational taxonomic unit (OTU) level, alpha diversity of microorganisms in the rhizosphere of plants was measured using observed OTU and Shannon index. The Shannon index and the observed OTU index showed that the cucJ group was significantly higher than the cuc group and the bulk soil was significantly higher than the cuc and cm3 groups at T1 (Wilcoxon rank sum test P < 0.05; **Figures 1A, B**). However, the Shannon index was significantly higher in the bulk group than in the cm3 group (Wilcoxon rank sum test P < 0.05), but the observed OTU index was not significantly different at T2 (Wilcoxon rank sum test P > 0.05; **Figures 1C, D**).

To clarify the differences in the bacterial communities of the different groups, the beta diversity of bacteria was visualized by PCoA based on the Bray-Curtis distance metric. The extremely significant differences in bacterial community composition between bulk soil and plant rhizosphere (PERMANOVA test; P = 0.000050 for T1, P = 0.00010 for T2; **Figures 1E, F**) suggested that plants influence bacterial communities. Also, PCoA analysis revealed a striking change in the composition of the bacterial communities of the cuc rhizosphere after infestation by RKN (PERMANOVA test; P = 0.0081; **Figure 1G**).

### 3.2 Changes in bacterial communities composition after inoculation with RKN

**Figure 2:**
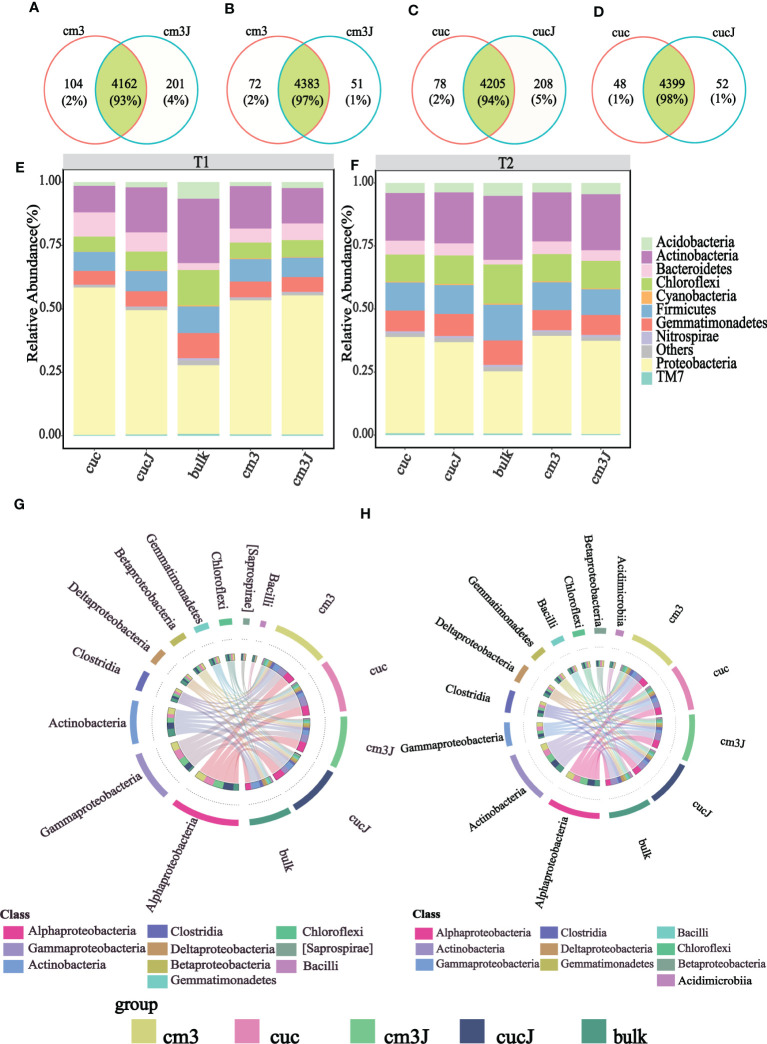
The cm3 and cm3J share the number of OTU species at T1 **(A)** and T2 **(B)** and cuc and cucJ share the number of OTU species at T1 **(C)** and T2 **(D)**. The relative abundance of major bacterial at T1 (phylum level; **(E)** and T2 (phylum level; **(F)** taxa present in the rhizosphere of different groups. Circos plot showing the taxonomical relative abundance of the top 10 bacterial microbiome at the class level at T1 **(G)** and T2 **(H)**. The thickness of each ribbon represents the relative abundance of bacterial assigned to different groups.

After RKN infestation, rhizosphere bacterial OTU species varied little, with 93% and 97% of the same OTU for cm3J and cm3 (**Figures 2A, B**), respectively, and 94% and 98% of the same OTU for cucJ and cuc (**Figures 2C, D**), respectively, in both periods. In order to gain insight into the taxonomic composition of rhizosphere bacterial community of different treatments, differences in the taxonomic composition of rhizosphere bacteria were compared at the phylum level (**Figures 2E, F**). The bacterial community at T1 was mainly composed of Proteobacteria, Actinobacteria, Firmicutes, Chloroflexi, Gemmatimonadetes, Bacteroidetes and Acidobacteria. At T1, the abundance of Acidobacteria and Nitrospirae increased in cm3J compared with cm3. Compared with the cuc group, the relative abundance of Actinobacteria, Acidobacteria, TM7, Nitrospirae and Cyanobacteria increased in the cucJ group. The dominant bacterial community at T2 was the same as in T1 period, but no differences in composition ratios were observed. Only the relative abundance of Cyanobacteria increased in cucJ than in cuc. Obviously, the taxonomic ratios of the bacterial community between bulk soil and plant rhizosphere were quite different in each period.

Next, the differences in the content of the top 10 bacteria in relative abundance at the class level were compared across the groups (**Figures 2G, H**). At T1, Gammaproteobacteria abundance appeared to decrease, but Actinobacteria and Bacilli increased to varying degrees in cucJ compared to cuc. Conversely, cm3J was more abundant in Betaproteobacteria but less abundant in Actinobacteria and Bacilli compared to cm3. At T2, Gammaproteobacteria and Betaproteobacteria decreased and Deltaproteobacteria increased in cucJ. Otherwise, Actinobacteria increased and Gammaproteobacteria, Chloroflexi, Betaproteobacteria and Bacilli decreased in cm3J.

### 3.3 Differences in rhizosphere bacteria of resistant and susceptible plants after inoculation with RKN

**Figure 3:**
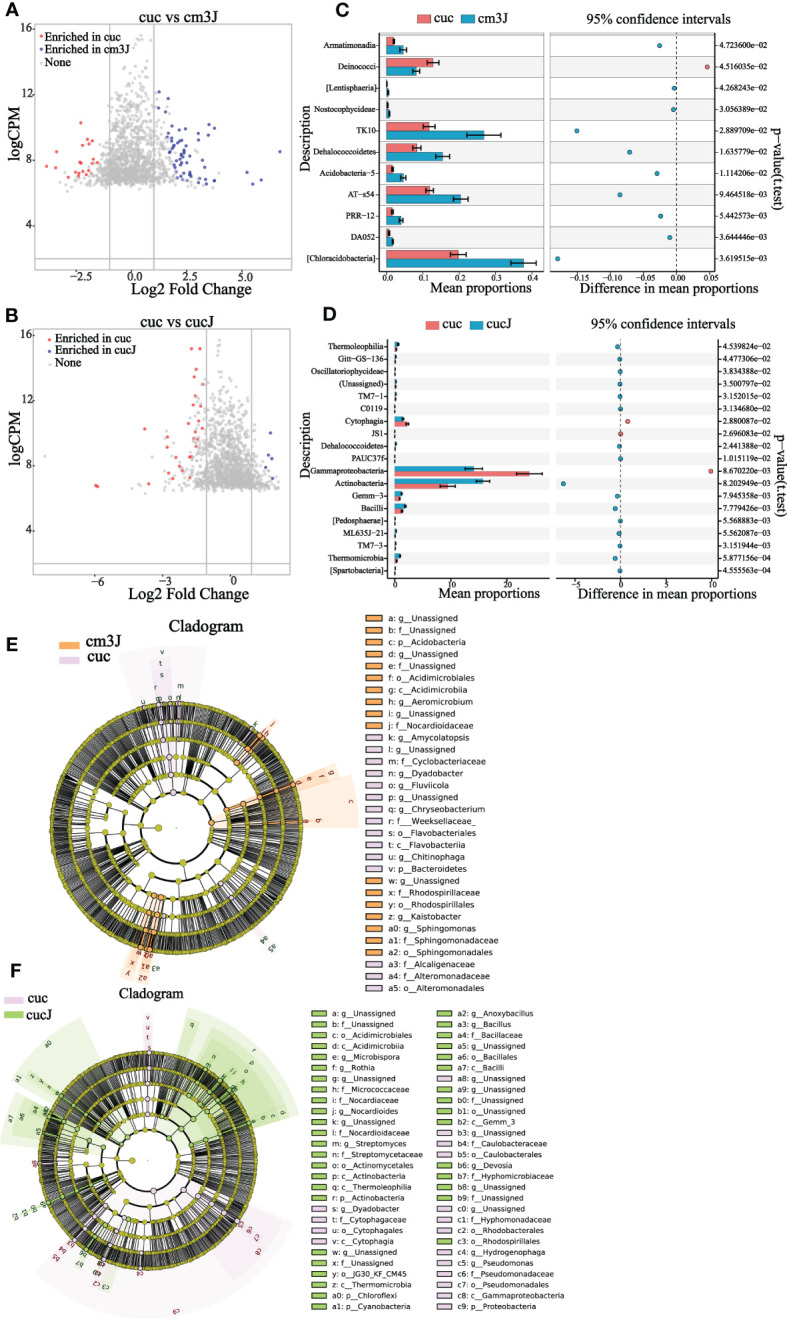
Cm3J **(A)** and cucJ **(B)** enriched OTU species compared to cuc at T1. STAMP analysis demonstrates differential enrichment of bacteria (class level) in the cm3J **(C)** and cucJ **(D)** at T1. Cladogram showing the bacteria phylogenetic structure of cm3J **(E)** and cucJ **(F)** with cuc respectively at T1.

The difference in the rhizosphere enrichment of bacterial OTU in cm3J (**Figure 3A**; **Supplementary Figure S1A**) and cucJ (**Figure 3B**; **Supplementary Figure S1B**) compared with cuc, respectively. The cm3J enriched a quantity of OTU relative to cuc in both periods, while cucJ only enriched a small amount of OTU in T1 period. Next, we compared the top 20 bacteria with differences in abundance in cm3J and cucJ with cuc, respectively. Specifically, the abundance of Armatimonadia, TK10, Dehalococcoidetes, Acidobacteria−5, AT−s54, PRR−12, DA052 and Chloracidobacteria was significantly higher in cm3J than in cuc (t.test, P < 0.05; **Figure 3C**) at T1. Only the abundance of Actinobacteria was significantly higher in cucJ than in cuc (t.test, P < 0.05; **Figure 3D**). Cm3J recruited more Chloroflexi and Alphaproteobacteria, while cucJ recruited more Thermomicrobia (**Supplementary Figure S1C, D**) at T2.

Compositional differences were assessed by calculating the linear discriminant analysis effect size (LEfSe) scores at different levels to find out the changes in the relative abundance of bacteria in resistant and susceptible plants after infection with RKN, respectively (**Supplementary Figure S2**). Under the condition of LDA threshold ≥ 3.0, cm3J was enriched Acidobacteria, Nocardioidaceae, Rhodospirillales, Sphingomonadales, Acidimicrobiia compared with cuc (**Figure 3E**) at T1. And cucJ was enriched with a considerable amount of bacteria compared to cuc including Bacilli, Cyanobacteria, Chloroflexi, Actinobacteria, Rhodospirillales, Hyphomicrobiaceae and Gemm_3 (**Figure 3F**). Thus, it can be found that cucJ enriched more bacterial after inoculation with RKN. Cm3J was mainly enriched with Chloroflexi, Actinobacteria, *Stenotrophomonas* and Alphaproteobacteria (**Supplementary Figure S1E**) at T2. Furthermore, there was only little difference between cucJ and cuc compared to rhizosphere bacteria. CucJ was mainly enriched with Thermomicrobia and Microbacteriaceae (**Supplementary Figure S1F**).

### 3.4 Changes in the co-occurrence network of bacterial communities

**Figure 4:**
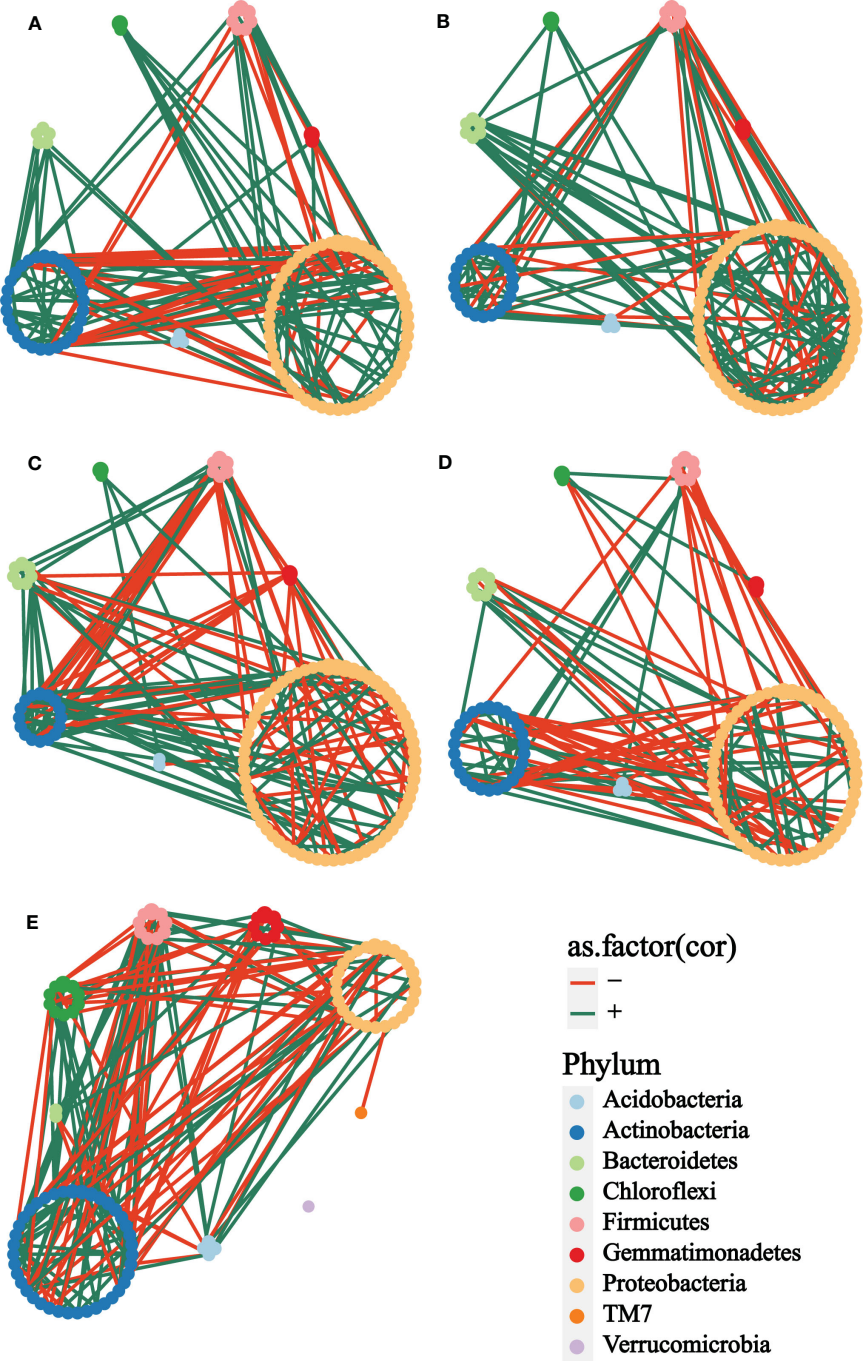
Co-occurrence network analysis of the rhizosphere microbial communities of different groups at T1. **(A)** cm3, **(B)** cuc, **(C)** cm3J, **(D)** cucJ, **(E)** bulk. The networks were colored based on the taxonomy taxa of bacteria at the phylum level. Edge s indicated correlations, which were divided into positive (green) or negative (red) correlations.

In exploring the interactions of rhizosphere bacterial communities of plants with different treatments at different growth stages, co-occurrence networks were constructed to demonstrate the differences in rhizosphere bacterial interactions. Firstly, it was straightforward to see that the bacterial network of the bulk soil (**Figure 4E**) was obviously different from the other groups. The taxonomic composition of the network showed no apparent differences among the cm3 (**Figure 4A**), cuc (**Figure 4B**), cm3J (**Figure 4C**) and cucJ (**Figure 4D**) groups, with most nodes belonging to Proteobacteria, Actinobacteria, Bacteroidetes, Firmicutes, Gemmatimonadetes, Chloroflexi, and Acidobacteria, respectively. The network differences among the groups at T1 were greater than those at T2, so specific analyses were performed for the bacterial networks of the groups at T1.

From cm3J to cucJ, the network complexity gradually decreased. There were 148 edges in the cm3J network, while 115 edges in the cucJ. The average degree and relative.modularity of cm3J were 3.6098 and 1.3185, edge density and diameter were 0.0446 and 1.9494 respectively, while the average.degree and relative.modularity of cucJ were 2.5556 and 0.6200, edge density and diameter were 0.0287 and 2.8704, respectively. More importantly, the positive correlation accounted for 74.32% and the negative correlation accounted for 25.68% in cm3J, while the positive and negative correlations in cucJ were 60.00% and 40.00%, respectively (**Supplementary Table S1**). Hub nodes were nodes that were more connected with other nodes in the network, and hub microorganisms were those that were closely connected with other microorganisms. The top five OTU with hub_score in the cm3J belonged to Firmicutes, Actinobacteria and Proteobacteria, respectively. Similarly, they belonged to Actinobacteria and Firmicutes respectively in the cucJ (**Supplementary Figure S3**).

### 3.5 Screening for RKN resistant bacteria

**Figure 5:**
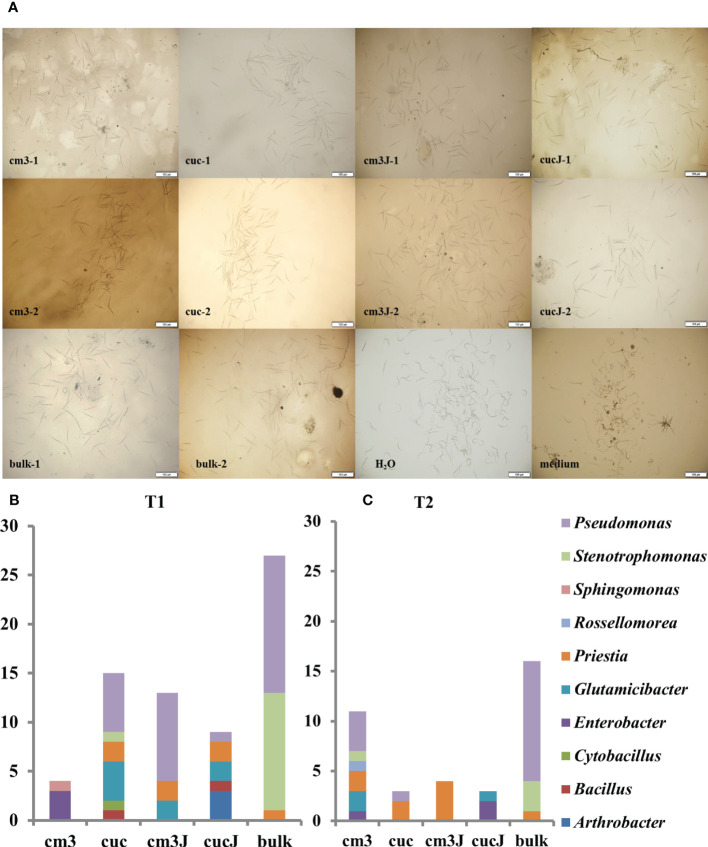
Effectiveness of antagonistic bacteria in killing nematodes **(A)**. Statistics of antagonistic bacteria genus in different groups at T1 **(B)** and T2 **(C)**. -1: T1; -2: T2.

105 antagonistic bacteria were yielded screening of bacteria resistant to *M. incognita* using R2A and LB medium and classified in 10 genus (**Figure 5A**), of which 44.76% *Pseudomonas*, 15.24% *Priestia*, 16.19% *Stenotrophomonas* and 10.48% *Glutamicibacter* (**Supplementary Table S2**). More antagonistic bacteria were obtained in the cm3J than in the cucJ at T1, and both were less abundant than in the bulk soil. However, the cm3J obtained fewer species of antagonistic bacteria than the cucJ and the bulk soil (genus level). Similar screening results to T1 (**Figure 5B**) were showed at T2 (**Figure 5C**). Others, more antagonistic bacteria were obtained in cm3J and cucJ at T1 than T2. Notably, the antagonistic bacteria in the cm3J group were *Pseudomonas*, *Glutamicibacter*, and *Priestia*. The *Arthrobacter*, *Bacillus*, *Glutamicibacter*, *Priestia*, *Pseudomonas* and *Enterobacter* were identified in the cucJ group.

## 4 Discussion

### 4.1 Rhizosphere bacterial communities responded to RKN invasion in the early stages

Changes in soil microbial communities were the most important biological factors influencing the occurrence of soil diseases (Kloeppe et al., 1999; Larkin, 2008). In previous studies, the occurrence of RKN disease was found to be closely related to the interactions between soil microbial communities (Echeverrigaray et al., 2010). Plants infected with RKN responded with resistance by releasing many compounds into the rhizosphere (Čepulytė et al., 2018; Oota et al., 2020; Rutter et al., 2022). Nutrients and metabolites were released through root cells fed by nematodes through the symplast, thereby altering the composition of root exudates (Tian et al., 2015), which in turn may affect the microbial community. Similarly in this study, RKN invaded the plant roots and the rhizosphere bacterial species diversity and community composition changed. Moreover, changes in the rhizosphere bacterial community were more pronounced in the early stages of plant-root knot nematodes interaction, which was consistent with the findings of other studies (Poll et al., 2007). In the early stages of RKN infestation, the species diversity of rhizosphere bacteria increased in cucJ and cm3J, and the composition of the cucJ rhizosphere bacterial community was apparently altered. In addition, there were obvious differences in the rhizosphere bacterial co-occurrence network. These changes may be the result of a combination of attacks by RKN on rhizosphere microbial structures and the plant response in the early stages.

### 4.2 Stabilization of rhizosphere bacterial community structure to suppress RKN

In the study of rhizosphere bacterial community structure, it was found that the rhizosphere network was substantially more complex than the bulk soil, indicating that rhizosphere had greater potential for interaction and ecological niche sharing (Shi et al., 2016). Although the species diversity and community composition of both cm3J and cucJ rhizosphere bacterial communities changed in the face of RKN invasion, the cm3J community structure was more stable with only small changes and no obvious changes in species diversity and community composition, as well as fewer bacterial enrichment species. The homeostasis of soil microorganisms as a dynamic component of the plant-soil system was extremely important for the control of soil-borne diseases. Cooperative and competitive interactions between microbial species and the modularity of the network influence the stability of the community (Faust and Raes, 2012; Coyte et al., 2015). Studies on the rhizosphere microbial community showed that the bacterial community structure of resistant genotypes was more stable than that of sensitive genotypes (Fu et al., 2019; Dong et al., 2021). Likewise, the bacterial community structure of cm3J was more stable than that of cucJ in this study.

The edges connecting two nodes representing different units in a microbial network indicate close associations between the abundance of these units in the whole sample, often interpreted as biological interactions. A great number of microbial interactions have also been confirmed experimentally in the *Arabidopsis* root community (Durán et al., 2018). In this study, the interactions and positive correlations between rhizosphere bacteria in cm3J were stronger, forming a more complex co-occurrence network. This indicated that the ecological interactions of cm3J rhizosphere dominant bacteria were more positive (Zhang et al., 2018; Lian et al., 2019). Elsewhere, the network complexity (Wagg et al., 2019) and hub taxa in supporting ecosystem function were important (Toju et al., 2018). In contrast, the rhizosphere bacterial community of cucJ was more susceptible to disturbance. When infested by RKN, the cuc rhizosphere bacterial network transmitted the environmental disturbance to the whole network with a short time, in turn destabilizing the network structure. Network inference can provide insights into microbial community composition, but theoretical studies of the effects of some network properties on ecosystem stability still require experimental evidence (Faust, 2021).

After that, we found a close positive correlation between Bacteroidetes and Proteobacteria in the cm3J network. Among the soil microbiota, the Bacteroidetes tended to be a dominant phylum due to their ability to secrete a variety of carbohydrate-active enzymes (CAZymes) that targeted highly variable glycans in the soil (Larsbrink and McKee, 2020). Bacteroidetes were abundant pathogen suppressor members of the plant microbiome and contributed to rhizosphere phosphorus mobilization (Lidbury et al., 2021). Proteobacteria were also abundant, as typically observed in soil libraries (Janssen, 2006). And multiple positive interactions of Pseudomonadales and Rhizobiales with other bacteria, such as Sphingomonadales. The probiotic *Pseudomonas* (Gammaproteobacteria, Pseudomonadales) was versatile in terms of plant hosts, soil habitat and improving plant stress response (Kim and Anderson, 2018). The most known effects of *Pseudomonas* were the protection of plants from fungal diseases and the improvement of plant yield, as well as the recent discovery of interesting aspects regarding insecticidal activity (Ruffner et al., 2013). *Rhizobia* were a group of soil-borne bacteria that had the ability to fix atmospheric nitrogen for plant growth and promoted root growth (Masson-Boivin et al., 2009; Poupin et al., 2016; Garrido-Oter et al., 2018). *Sphingomonas* was the main group of rhizosphere and endophytic bacteria with multifaceted functions ranging from remediation of environmental pollution to production of highly beneficial phytohormones involved in rhizosphere remediation of organic matter (Zhang et al., 2013; Feng et al., 2019; Asaf et al., 2020). Additionally, Sphingomonadaceae, Brucellaceae, and Bartonellaceae were also closely associated with other rhizosphere microorganisms. Brucellaceae and Bartonellaceae were associated with nematodes carriage (Bowman, 2011). In summary, the more positive correlation between probiotics and the close interaction with RKN-associated bacteria may be a reason for the resistance of cm3 to RKN infestation.

### 4.3 Recruiting beneficial bacteria

There was little change in bacterial species when RKN interacted with plant rhizosphere microorganisms; what changed was the abundance of some bacteria. In the early stage, the change in the abundance of bacteria in cucJ was an increase in Actinobacteria and Bacilli, a decrease in Bacteroidetes, Gammaproteobacteria and Betaproteobacteria. Actinobacteria played a role in soil nitrogen fixation, improving nutrient availability, and promoting the production of plant growth regulators (Bhatti et al., 2017). The Bacilli offered a number of advantages for their application in agricultural biotechnology, and some products based on Bacilli, especially *Bacillus*, have been marketed as microbial pesticides, fungicides or fertilizers (Perez-Garcia et al., 2011). Conversely, Actinobacteria and Bacilli decreased and Betaproteobacteria increased in cm3J. Cm3J was mainly enriched in Gammaproteobacteria and Betaproteobacteria. Differently, cucJ mainly recruited Actinobacteria, Saprospirae, Bacilli and Thermomicrobia.

In addition, Thiobacterales, iii1_15 and Kaistobacter may play a role in cm3J. In cucJ, Thermoleophilia, Streptomyces, Nocardioidaceae, Nocardioides, Micrococcaceae, Acidimicrobiales, Devosia, Gemm_3, Bacillaceae, Cyanobacteria and G30_KF_CM45 may play important roles. *Streptomyces* was the most abundant and important genus of actinomycetes. And *Streptomyces* had a beneficial symbiotic relationship with plants, promoting the nutrition and health of the latter (Pang et al., 2022). Nocardioidaceae can degrade a wide range of organic compounds, including aromatic and polyaromatic pollutants and toxic chemicals (Tóth and Borsodi, 2014). Plant-cyanobacterial interactions, as a beneficial symbiotic relationship, have long been demonstrated in rice growing areas. In addition, cyanobacteria may produce or secrete large amounts of biologically active compounds that have the ability to promote plant growth or may make plants more resistant to abiotic or biotic stresses (Bahareh et al., 2021). Most of the antagonistic bacteria screened were *Pseudomonas*, along with *Priestia*, *Stenotrophomonas*, and *Glutamicibacter*. A large number of *Pseudomonas* were screened in cm3J. *Stenotrophomonas* produced similar antibiotics and shared some enzymatic activities, which may make them attractive candidates for biological control of plant diseases and nematodes (Hayward et al., 2010).

We observed that RKN invasion caused recruitment and alteration of probiotic bacteria in both cm3J and cucJ. Cm3J was mainly enriched in the Proteobacteria and cuc was mainly enriched in the Actinobacteria and Bacilli. Moreover, the most abundant bacteria screened for antagonistic bacteria was *Pseudomonas* (Proteobacteria, Pseudomonadaceae). It was possible that *Pseudomonas* and other bacteria of the Proteobacteria played a part in RKN infestation of cm3J.

### 4.4 Hypothesizing the causes of differences in rhizosphere microbial communities

Root exudates of two crops may cause rhizosphere microbial differences. Root exudates include sugars, amino acids, organic acids, fatty acids, and secondary metabolites, which are important ways for plants to communicate with microorganisms and have a major influence on the composition of the rhizosphere microbiome (Bulgarelli et al., 2013; Sasse et al., 2018). Moreover, root exudates recruit microorganisms from the soil to the rhizosphere, where primary metabolites are mainly responsible for attraction and secondary metabolites are mainly responsible for screening the recruited microorganisms (Zhalnina et al., 2018). Furthermore, root exudates were strongly affected by plant species, developmental stage, root physiology, environment, soil type and stress type (Sasse et al., 2018). Beyond that, the assembly of microbial communities in plant roots also depends on microbial interactions (Bai et al., 2022). A study constructed a highly simplified maize SynCom, and single strain exclusion experiments on SynCom showed that no bacterial strains except Enterobacter cloacae caused SynCom collapse, indicating that Enterobacter cloacae was a key member in the community assembly process (Niu et al., 2017). In the case of cm3J and cucJ after infection with RKN in this study, it was possible that different root exudates and microbial interactions contributed to the differences in rhizosphere microbial communities.

## 5 Conclusion

In general, the interaction between RKN and rhizosphere bacteria was stronger in the early growth period, which provides a reference for the period of biocontrol. Secondly, a stable and well-connected rhizosphere bacterial community was positive for suppressing RKN infestation. Finally, *Pseudomonas* and other bacteria in the Proteobacteria in *Cucumis* crops showed clear changes in response to RKN invasion, which pointed the way for further research on biocontrol agents.

## Data availability statement

The 16S sequence datasets for this study can be found in the NGDC with accession number: PRJCA014808 (https://ngdc.cncb.ac.cn).

## Author contributions

BX and JL designed the experiment. ZM, YL, YY and JZ directed the experiment and the writing. XP and LS performed the experiment and processed the data. LS analysed the data and wrote the manuscript. JL revised the manuscript. All authors contributed to the article and approved the submitted version.
