# Supplementary material for: Variation and stability of rhizosphere bacterial communities of Cucumis crops in association with root-knot nematodes infestation
Source: Front Plant Sci. 2023 May 30;14:1163271. doi: 10.3389/fpls.2023.1163271 (PMC10266268; doi:10.3389/fpls.2023.1163271)
Supplement: Supplementary file 1 [file DataSheet_1.docx]

Supplementary Material

Variation and stability of rhizosphere bacterial communities of *Cucumis* crops in association with root-knot nematodes infestation

Liqun Song *, Xingxing Ping, Zhenchuan Mao, Jianlong Zhao, Yan Li, Yuhong Yang, Bingyan Xie and Jian Ling

*** Correspondence:** Jian Ling: lingjian@caas.cn, Bingyan Xie: xiebingyan@caas.cn

# Supplementary Figures and Tables

## Supplementary Figures


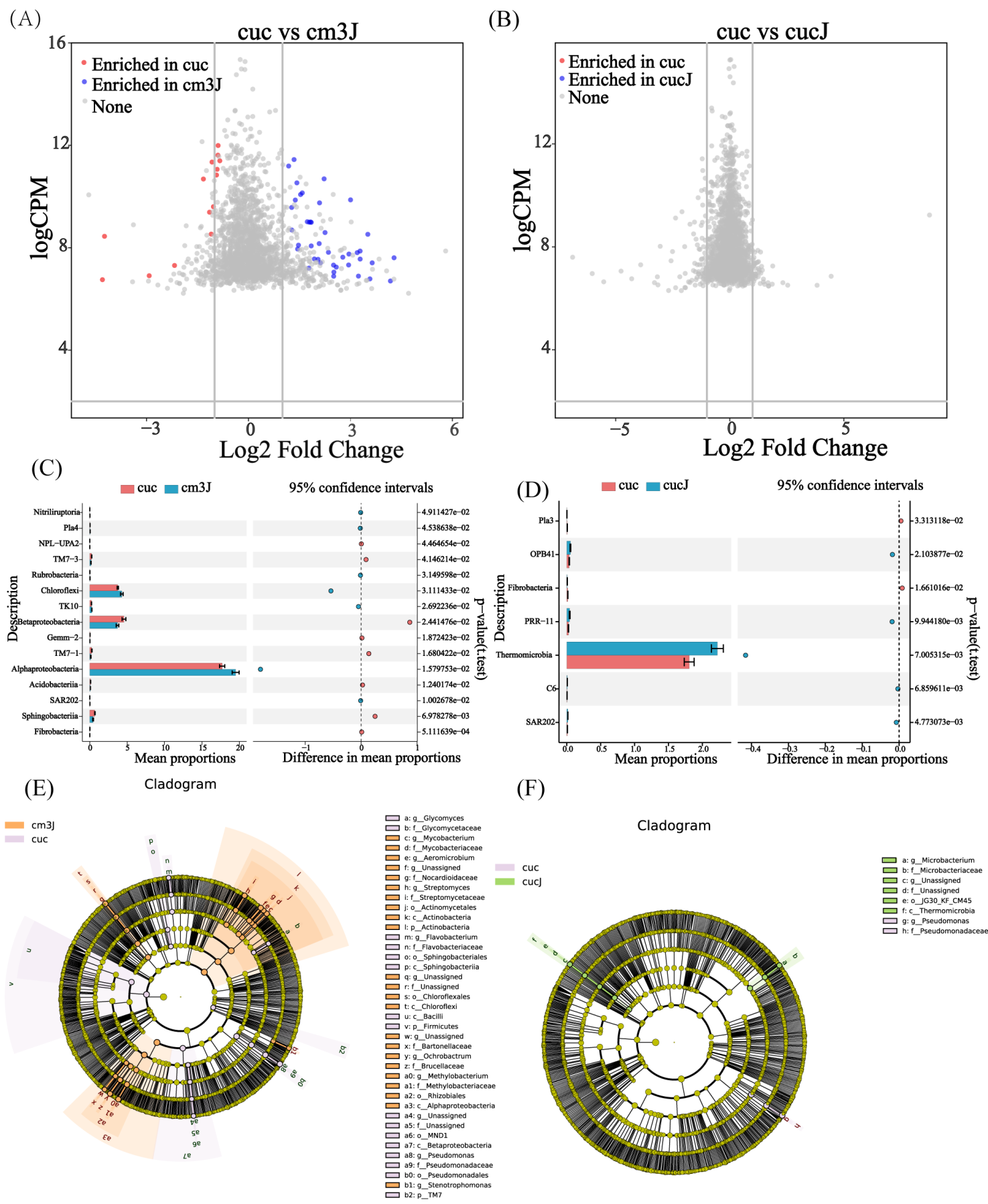


**Supplementary Figure 1.** Cm3J (A) and cucJ (B) enriched OTU species compared to cuc at T2. STAMP analysis demonstrates differential enrichment of bacteria (class level) in the cm3J (C) and cucJ (D) at T2. Cladogram showing the bacteria phylogenetic structure of cm3J (E) and cucJ (F) with cuc respectively at T2.


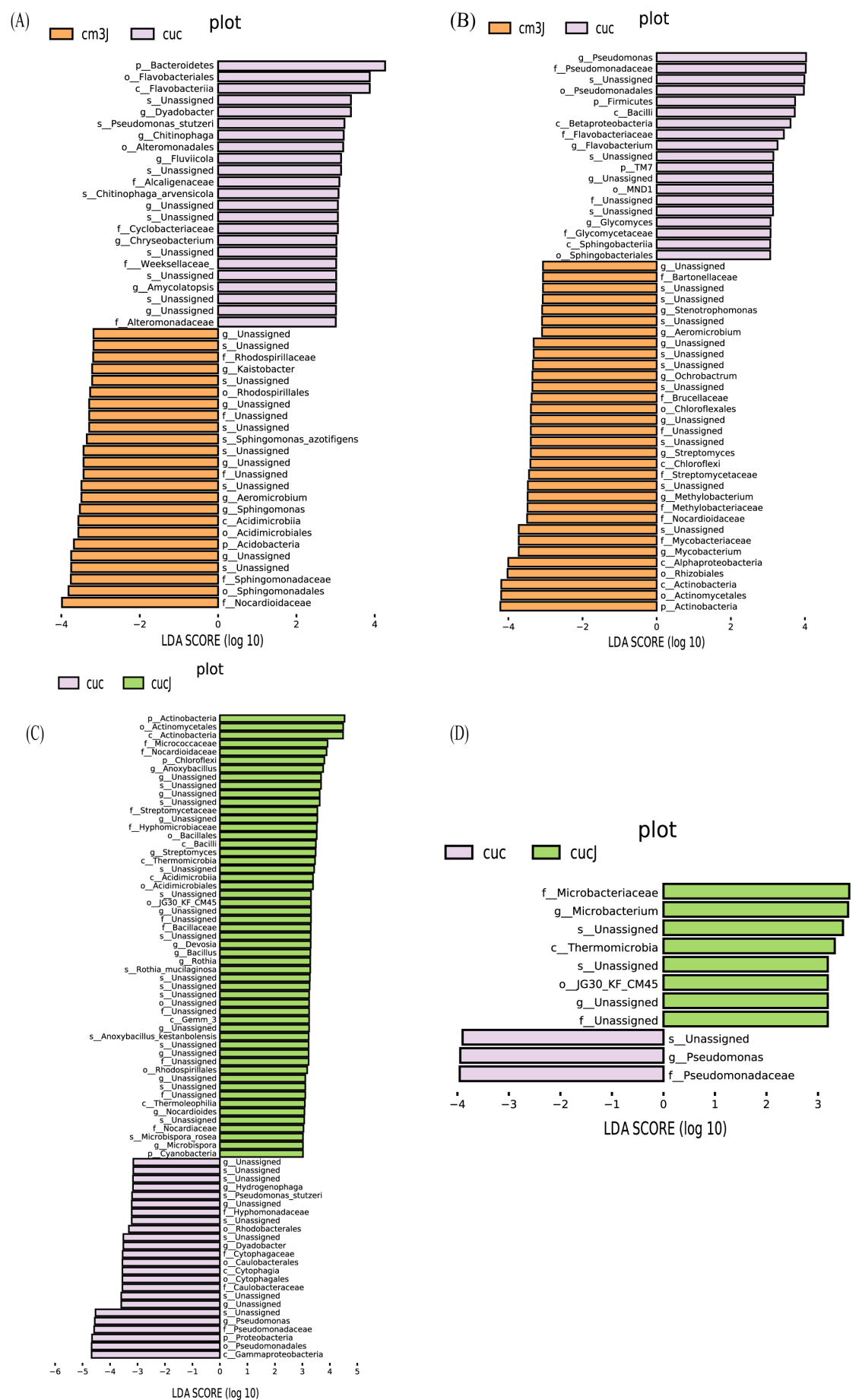


**Supplementary Figure 2.** Linear discriminant analysis effect size (LEfSe) analysis of differentially abundant (LDA threshold score ≥ 3.0) of bacteria at different levels of cm3J (A) and cucJ (C) with cuc respectively at T1, cm3J (B) and cucJ (D) with cuc respectively at T2.


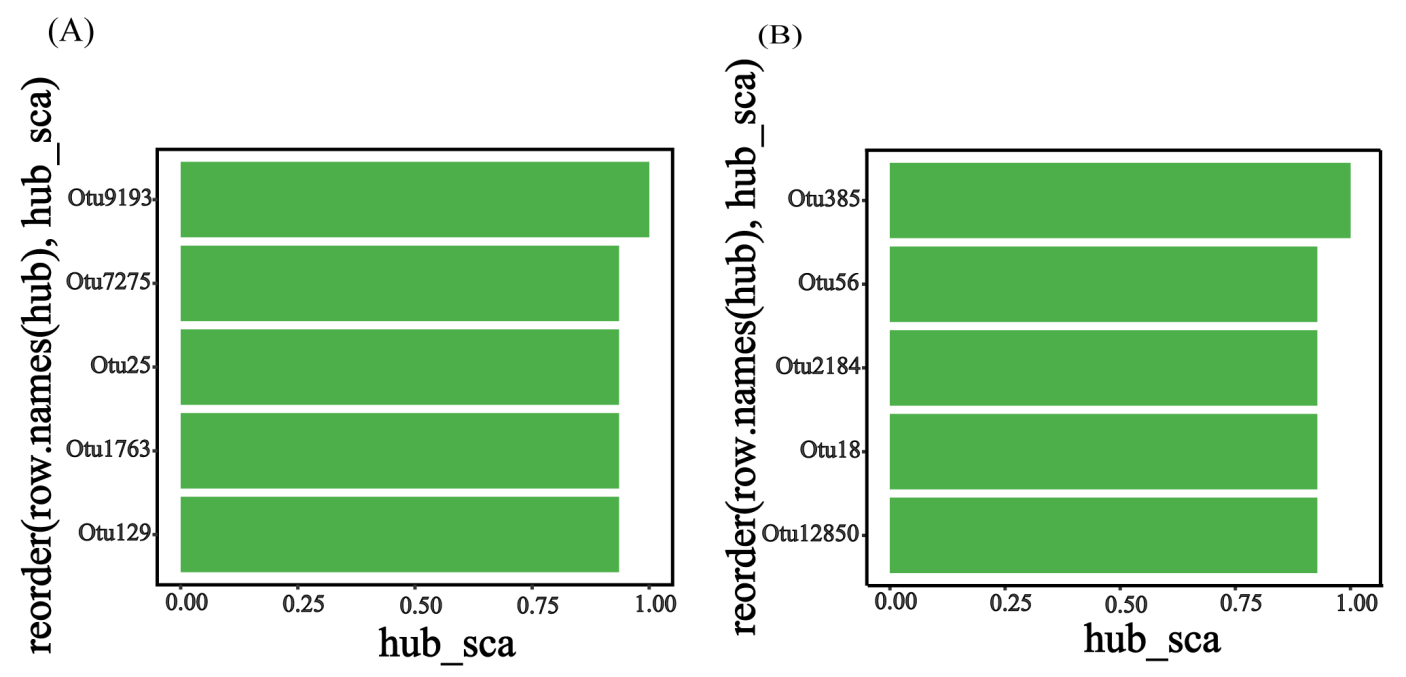


**Supplementary Figure 3.** The top five OTU with hub_score in the cm3J (A) and cucJ (B) at T1.

## Supplementary Tables

**Table 1. Co-occurrence network parameters for different groups at T1.**

|  | cucJ | cm3J | cm3 | cuc | bulk |
| --- | --- | --- | --- | --- | --- |
| num.edges(L) | 158 | 115 | 165 | 148 | 156 |
| num.pos.edges | 97 | 69 | 117 | 110 | 87 |
| num.neg.edges | 61 | 46 | 48 | 38 | 69 |
| num.vertices(n) | 82 | 90 | 92 | 82 | 84 |
| Connectance(edge_density) | 0.047576 | 0.028714 | 0.039417 | 0.044565 | 0.04475 |
| average.degree(Average K) | 3.853659 | 2.555556 | 3.586957 | 3.609756 | 3.714286 |
| average.path.length | 1.874126 | 1.153846 | 1.10929 | 1.045161 | 1.223881 |
| diameter | 3.846725 | 2.870412 | 2.816785 | 1.949359 | 1.949359 |
| mean.clustering.coefficient(Average.CC) | 0.934836 | 0.944444 | 0.976119 | 0.986765 | 0.957108 |
| no.clusters | 18 | 28 | 25 | 20 | 20 |
| centralization.degree | 0.075881 | 0.027466 | 0.048495 | 0.029509 | 0.063683 |
| centralization.betweenness | 0.023106 | 0.002008 | 0.002415 | 0.001223 | 0.004302 |
| centralization.closeness | 0.003969 | 0.000851 | 0.001087 | 0.000751 | 0.001416 |
| RM(relative.modularity) | 1.004176 | 0.619972 | 1.021087 | 1.318526 | 1.051368 |

**Table 2. Statistics on the number and species of antagonistic bacteria.**

| Time | T1 | | | | | T2 | | | | |  |
| --- | --- | --- | --- | --- | --- | --- | --- | --- | --- | --- | --- |
| Group | cm3 | cuc | cm3J | cucJ | bulk | cm3 | cuc | cm3J | cucJ | bulk | Total |
| *Arthrobacter* | 0 | 0 | 0 | 3 | 0 | 0 | 0 | 0 | 0 | 0 | 3 |
| *Bacillus* | 0 | 1 | 0 | 1 | 0 | 0 | 0 | 0 | 0 | 0 | 2 |
| *Cytobacillus* | 0 | 1 | 0 | 0 | 0 | 0 | 0 | 0 | 0 | 0 | 1 |
| *Enterobacter* | 3 | 0 | 0 | 0 | 0 | 1 | 0 | 0 | 2 | 0 | 6 |
| *Glutamicibacter* | 0 | 4 | 2 | 2 | 0 | 2 | 0 | 0 | 1 | 0 | 11 |
| *Priestia* | 0 | 2 | 2 | 2 | 1 | 2 | 2 | 4 | 0 | 1 | 16 |
| *Rossellomorea* | 0 | 0 | 0 | 0 | 0 | 1 | 0 | 0 | 0 | 0 | 1 |
| *Sphingomonas* | 1 | 0 | 0 | 0 | 0 | 0 | 0 | 0 | 0 | 0 | 1 |
| *Stenotrophomonas* | 0 | 1 | 0 | 0 | 12 | 1 | 0 | 0 | 0 | 3 | 17 |
| *Pseudomonas* | 0 | 6 | 9 | 1 | 14 | 4 | 1 | 0 | 0 | 12 | 47 |
| Total | 4 | 15 | 13 | 9 | 27 | 11 | 3 | 4 | 3 | 16 |  |
